# The influence of HMF and furfural on redox-balance and energy-state of xylose-utilizing *Saccharomyces cerevisiae*

**DOI:** 10.1186/1754-6834-6-22

**Published:** 2013-02-15

**Authors:** Magnus Ask, Maurizio Bettiga, Valeria Mapelli, Lisbeth Olsson

**Affiliations:** 1 Department of Chemical and Biological Engineering, Industrial Biotechnology, Chalmers University of Technology, SE-41296, Gothenburg, Sweden

**Keywords:** Lignocellulosic ethanol, HMF, Furfural, Inhibitors, Redox metabolism, Energy metabolism

## Abstract

**Background:**

Pretreatment of biomass for lignocellulosic ethanol production generates compounds that can inhibit microbial metabolism. The furan aldehydes hydroxymethylfurfural (HMF) and furfural have received increasing attention recently. In the present study, the effects of HMF and furfural on redox metabolism, energy metabolism and gene expression were investigated in anaerobic chemostats where the inhibitors were added to the feed-medium.

**Results:**

By cultivating the xylose-utilizing *Saccharomyces cerevisiae* strain VTT C-10883 in the presence of HMF and furfural, it was found that the intracellular concentrations of the redox co-factors and the catabolic and anabolic reduction charges were significantly lower in the presence of furan aldehydes than in cultivations without inhibitors. The catabolic reduction charge decreased from 0.13(±0.005) to 0.08(±0.002) and the anabolic reduction charge decreased from 0.46(±0.11) to 0.27(±0.02) when HMF and furfural were present. The intracellular ATP concentration was lower when inhibitors were added, but resulted only in a modest decrease in the energy charge from 0.87(±0.002) to 0.85(±0.004) compared to the control. Transcriptome profiling followed by MIPS functional enrichment analysis of up-regulated genes revealed that the functional group “Cell rescue, defense and virulence” was over-represented when inhibitors were present compared to control cultivations. Among these, the ATP-binding efflux pumps *PDR5* and *YOR1* were identified as important for inhibitor efflux and possibly a reason for the lower intracellular ATP concentration in stressed cells. It was also found that genes involved in pseudohyphal growth were among the most up-regulated when inhibitors were present in the feed-medium suggesting nitrogen starvation. Genes involved in amino acid metabolism, glyoxylate cycle, electron transport and amino acid transport were enriched in the down-regulated gene set in response to HMF and furfural. It was hypothesized that the HMF and furfural-induced NADPH drainage could influence ammonia assimilation and thereby give rise to the nitrogen starvation response in the form of pseudohyphal growth and down-regulation of amino acid synthesis.

**Conclusions:**

The redox metabolism was severely affected by HMF and furfural while the effects on energy metabolism were less evident, suggesting that engineering of the redox system represents a possible strategy to develop more robust strains for bioethanol production.

## Background

Utilization of lignocellulosic biomass for fuel ethanol production requires the raw material to be pretreated. The pretreatment aims at altering the structure of the material so its susceptibility towards (enzymatic) hydrolysis is increased. Due to the recalcitrant nature of lignocellulosic materials, harsh conditions have to be used, which result in production of several by-products that can have inhibitory effects on microbial metabolism [1]. Two of these compounds, 5-hydroxymethyl furfural (HMF) and 2-furaldehyde (furfural), have been subjects of extensive investigation due to their negative effects on microbial physiology, as reviewed in [2]. They are formed as dehydration products of hexoses and pentoses, respectively, and have been shown to inhibit cell growth, decrease ethanol productivity, induce DNA damage and inhibit several enzymes in glycolysis, thus posing a serious challenge for the feasibility of lignocellulosic ethanol production [3-5]. As it is advantageous to produce as high ethanol titer as possible (at least 40–50 g L^-1^[6]) to make the distillation energy efficient, the production process has to be pushed towards higher solid loadings, which leads to higher inhibitor concentrations. More robust ethanol producing microorganisms are thus needed to cope with such process constraints and hence, there is a need to increase the understanding of the physiological responses to HMF and furfural in order to increase strain robustness.

*Saccharomyces cerevisiae* has been shown to be capable of converting HMF and furfural to less inhibitory compounds *in situ,* as long as the concentrations are below lethal levels. Under anaerobic conditions, HMF and furfural are mainly converted to their corresponding alcohols, furan dimethanol and furfuryl alcohol, respectively, while during fully respiratory metabolism, furfural is converted to furoic acid [7-9]. The proposed mechanism for the intracellular conversion of HMF and furfural has been hypothesized to be NAD(P)H-dependent reduction and NAD(P)^+^-dependent oxidation by oxidoreductases under anaerobic conditions and fully respiratory metabolism, respectively [9,10]. Overexpression of several oxidoreductases such as *ADH6, ADH7* and *ZWF1* has shown to improve HMF and furfural detoxification in terms of either decreasing the lag-phase of cell growth by 30% or increasing the specific HMF and furfural conversion rate 3.5-fold compared to the respective wild-type strains [11-13]. Furthermore, the involvement of NAD(P)H-dependent oxidoreductive reactions for furan aldehyde conversion has also been shown in bacteria [14,15]. Hence, the availability of reduced redox co-factors seems to be important for furan aldehyde detoxification.

Under anaerobic conditions, NADH is mainly generated via reduction of NAD^+^ in glycolysis and in biosynthesis of amino acids [16]. Glycolysis is redox neutral, which means that the redox balance is maintained by the reduction of acetaldehyde to ethanol, thereby regenerating NAD^+^. The NADH generated in biosynthetic pathways is reoxidized by formation of glycerol [17]. The majority of NADPH is produced via reduction of NADP^+^ in the oxidative part of the pentose phosphate pathway (PPP), and the formed NADPH is mainly utilized in biosynthetic reactions [18]. Both NAD(H) and NADP(H) are highly interconnected within the metabolic network, meaning that perturbations in the levels of these compounds can have a big impact on metabolism [19]. In fact, NAD^+^, NADH, NADP^+^ and NADPH were included in 78, 65, 86 and 78 reactions, respectively, in a reconstructed metabolic network of *S. cerevisiae*[20]. In the present study, we focused on the quantification of the intracellular concentrations of NAD(P)^+^ and NAD(P)H, since in spite of the fact that the conversion of HMF and furfural to less inhibitory compounds is thought to proceed through NAD(P)H dependent reactions, there are surprisingly few reports on the actual impact of furan aldehyde stress on the intracellular levels of the redox factors*.*

Besides intracellular redox balance, stress also affects the energy metabolism as many stress responsive processes are dependent on ATP availability [21]. Firoved et al. [22] have reported that furfural addition to the feed of a glucose-limited chemostat did not result in any changes in the energetics in terms of maintenance energy of *S. cerevisiae*. On the other hand, several reports have suggested that furfural and HMF decrease the specific sugar uptake rates and specific ethanol production rate [9,23], factors highly correlated with energy generation in the form of ATP. Similar to the redox co-factors, the adenonucleotides ATP, ADP and AMP are participating in numerous intracellular reactions and perturbations in the intracellular concentrations of these can have major effects on metabolism [24]. It has been shown that ATP, ADP and AMP can function as allosteric regulators of the glycolytic flux by acting as effectors of phosphofructokinase and pyruvate kinase [25]. Changes in the levels of adenonucleotides following addition of HMF and furfural were considered in this work, in order to investigate how furan aldehydes affect the energy balance of the cells.

A large amount of data on the physiological responses of *S. cerevisiae* to HMF and furfural exist in literature. Nonetheless, a wide array of not always comparable cultivation conditions and experimental setups have been used [2]. Two different approaches have generally been utilized to study the effect of HMF and/or furfural on cellular metabolism. Either the inhibitors have been included in the cultivation media initially in batch cultures or they have been pulsed during the exponential or stationary growth phases [8,26-28]. Taherzadeh et al. [26] pulsed anaerobic batch cultivations with 2 or 4 g L^-1^ of furfural and observed decreases in specific growth rate, carbon dioxide evolution rate (CER), ethanol production rate and glycerol production rate. In other reports the furan aldehydes have been added to the feed-medium of continuous cultures [9,13,23,29]. Horvath et al. [29] added furfural at different concentrations to the feed-medium of glucose-limited anaerobic chemostats and observed increases in ethanol and biomass yield, while the glycerol yield decreased with increasing concentration of furfural. Almeida et al. [23] supplied 2 gL^-1^ HMF initially in an anaerobic batch cultivation, which resulted in decreased xylose consumption rate, decreased xylitol yield and increased acetate yield compared to the control cultivation without inhibitors. In the same study, by having 2 g L^-1^ HMF present in the feed-medium of an anaerobic chemostat cultivation, decreases in specific glucose uptake rate, xylose uptake rate, ethanol production rate, glycerol production rate and xylitol production rate were observed. Since the choice of experimental setup clearly has a significant impact on the metabolic responses, it is difficult to elaborate an unequivocal explanation of the physiological response to HMF and furfural from the available data.

In the present study, the impact of HMF and furfural on the anaerobic physiology of *S. cerevisiae* was studied by subjecting the cells to a continuous stress by including the inhibitors in the feed-medium of carbon-limited anaerobic chemostats. In this setup, the cells are expected to adapt to the stress, which means that only genes required for growth in the presence of HMF and furfural are likely to be differentially expressed, in contrast to pulsed cultivations where an instantaneous and transient stress response is expected [30,31]. To investigate the identity of these genes, transcriptome profiling was performed in the presence or absence of HMF and furfural in the feed-medium. An advantage of studying transcriptomic responses in continuous culture is that the specific growth rate can be held constant, as many genes responsive to stressful conditions have shown to be differentially expressed upon changes in specific growth rate [32]. Since most of the lignocellulosic hydrolysates contain xylose, and because xylose fermentation is a prerequisite to make lignocellulosic ethanol economically feasible, a xylose-fermenting yeast strain was used and both glucose and xylose served as carbon and energy sources. The strain used in the present study, VTT C-10883, is engineered with the xylose reductase/xylitol dehydrogenase (XR/XDH) pathway from *Scheffersomyces stipitis*. This is to the best knowledge of the authors, the first report on the quantification of intracellular redox and energy factors at sub-lethal concentrations of HMF and furfural.

## Results

A thorough understanding of the effects of HMF and furfural on yeast metabolism is required to generate potential metabolic engineering strategies that can improve strain robustness. Since lignocellulosic hydrolysates contain a mixture of the furan aldehydes HMF and furfural, and the proposed mechanism of detoxification is similar for both compounds [2], both were added to the feed-medium of carbon-limited chemostats. In most studies where the physiological responses to furan aldehydes have been studied, high concentrations of inhibitors have been pulsed thereby arresting cell growth. By having the inhibitors in the feed-medium, a constant stress can be applied and cell growth is maintained.

### Targeting the sub-lethal concentrations of HMF and furfural

Including HMF and furfural in the feed-medium requires a fine-tuning of the respective concentrations since both HMF and furfural inhibit cell growth. In the present study, the furaldehyde concentrations were chosen so that the cells were as challenged as possible, but still allowing growth at the selected dilution rate of 0.1 h^-1^. When steady-state had been reached, HMF and furfural were added to the feed-medium reservoir. An iterative approach was applied to find the maximum concentrations of the inhibitors and the molar HMF:furfural ratio was kept constant at 2.3:1, a value common for spruce hydrolysates, according to [33]. As tolerance against inhibitors partly depends on cell density, the inhibitor concentrations were normalized to the cell density in the reactors. Initially, 0.7 mmol HMF (g DW)^-1^ and 0.3 mmol furfural (g DW)^-1^ were added. If no change in carbon dioxide evolution rate (CER) was observed, the concentrations of inhibitors were increased. If wash-out occurred, as indicated by a steady drop in CER, the concentrations of inhibitors were lowered. After five consecutive steps of iteration, the maximum concentrations of HMF and furfural that allowed growth at 0.1 h^-1^ were found to be 5.7 mmol HMF (g DW)^-1^ and 2.4 mmol furfural (g DW)^-1^ (corresponding to 10.3 mM HMF and 4.2 mM furfural in the feed, respectively) for the particular strain used in the experiments. A thorough physiological characterization was then performed at the selected inhibitor concentrations.

### Physiological characterization at sub-lethal concentrations of HMF and furfural

In order to characterize the change in physiology of the xylose-consuming yeast strain VTT C-10883 in the presence of furan aldehydes, chemostat cultivations with 20 g L^-1^ glucose and 50 g L^-1^ xylose were performed. When steady-state was reached the feed-medium was step-changed by including 5.7 mmol HMF (g DW)^-1^ and 2.4 mmol furfural (g DW)^-1^ in the feed-medium. Samples were taken for cell dry weight, extracellular metabolites, NAD(P)(H), ATP, ADP, AMP and gene expression analysis after a new steady-state was reached. A cultivation without inhibitors served as control. Specific consumption rates of glucose and xylose and specific production rates of selected extracellular metabolites are shown in Table 1. Glucose and xylose were co-consumed and it was found that approximately 95% of the glucose was consumed in the two conditions, while a considerable amount of residual xylose was left: only 18% of the xylose was consumed in both conditions. Moreover, the steady-state glucose concentration was higher than reported before for carbon-limited chemostats using only glucose as carbon source, where it usually is in the order of <0.5 g L^-1^[34]. The relatively high steady-state concentrations of residual glucose in both the presence and absence of inhibitors could be an effect of a saturated transport system, where the high concentration of xylose in the fermentation broth competitively inhibits glucose uptake. A low amount of furfural was detected in the medium, whereas 3.42 mM residual HMF was found in the fermentation broth at steady-state. Consequently, only 67% of the HMF was further converted, whereas close to all furfural in the feed was converted. The specific production rates of ethanol and CO_2_ decreased significantly (p < 0.01) in the presence of inhibitors, whereas the specific xylitol production rate increased (p < 0.01). No significant differences were observed in the specific production rates of glycerol, pyruvate and succinate between step-changed and control cultivations. Interestingly, the specific glucose and xylose consumption rates decreased by 10.9 (±2.3)% and 8.6 (±0.2)%, respectively, when HMF and furfural were present in the feed-medium. Moreover, the biomass yield increased by 10.2 (±3.7)% in the presence of inhibitors, indicating that an increased amount of carbon was redistributed to biomass production.

**Table 1:** Physiological data from anaerobic chemostats with and without HMF and furfural added to the feed-medium

	**Specific production rates (c-mmol g DW**^**-1**^ **h**^**-1**^**)**												
	**Ethanol**	**Glycerol**	**CO**_**2**_	**Acetate**	**Xylitol**	**Pyruvate**	**Succinate**	**Glc uptake**	**Xyl uptake**	**Biomass yield (g c-mol**^**-1**^**)**	**Residual HMF (mmol)**	**Residual furfural (mmol)**	**Carbon recovery**
Control	21.4 ± 0.8	5.37 ± 0.89	14.8 ± 0.8	0.283 ± 0.083	5.77 ± 0.17	0.078 ± 0.011	0.167 ± 0.001	36.6 ± 1.2	18.0 ± 0.3	1.81 ± 0.06	-	-	0.95 ± 0.03
Inhibitors	19.3 ± 0.4	4.91 ± 0.28	12.7 ± 0.5	0.256 ± 0.046	6.48 ± 0.36	0.069 ± 0.002	0.184 ± 0.029	31.9 ± 1.6	17.0 ± 1.0	2.06 ± 0.06	3.42 ± 0.15	0.021 ± 0.003	0.98 ± 0.02

The numbers reported are means from three individual bioreactors ± standard deviation.

### Growth in the presence of HMF and furfural drains the cells of reductive power

The redox carriers NAD(H) and NADP(H) are generally reported to play a role in the enzymatic reduction of HMF and furfural to their corresponding alcohols under anaerobic conditions [13,23,28]. Perturbations in the redox balance can have severe effects on metabolic pathways, since these compounds are involved in a large number of intracellular reactions. In order to quantify the change in the redox balance after including HMF and furfural in the feed-medium, the redox co-factors NAD(P)^+^ and NAD(P)H were measured at steady-state in stressed and non-stressed cells. Since these compounds are known to have short turnover time in the cell and respond quickly to changes in environment [35], the metabolism was stopped instantly after sampling by quenching in methanol at −40°C. Addition of HMF and furfural to the feed-medium caused a decrease in the intracellular steady-state level of NADH from 0.48 (±0.08) μmol (g DW)^-1^ to 0.20 (±0.06) μmol (g DW)^-1^, while the intracellular steady-state concentration of NADPH decreased from 0.55 (±0.17) μmol (g DW)^-1^ to 0.14 (±0.01) μmol (g DW)^-1^, compared the to feed-medium without inhibitors (Table 2). The ratio between reduced and oxidized co-factors is thought to play a major role in metabolism since several enzymes are regulated by this ratio [36]. The catabolic and anabolic reduction charges relate the reduced co-factor concentrations to the total pools of NAD(H) and NADP(H), respectively. The catabolic and anabolic reduction charges (see methods section) were found to be significantly lower in stressed cells. In the absence of inhibitors, the catabolic reduction charge was 0.13 (±0.005), whereas in the presence of inhibitors it was found to be 0.08 (±0.002). The anabolic reduction charge was estimated to 0.46 (±0.11) in the absence and 0.27 (±0.02) in the presence of HMF and furfural in the feed-medium (Table 2). These findings indicate that the cells are drained of reductive power in the presence of HMF and furfural.

**Table 2:** Intracellular concentrations of redox co-factors at steady-state from chemostat cultivations with and without HMF and furfural added to the feed-medium

	**[NAD**^**+**^**]**	**[NADH]**	**Catabolic reduction charge**	**[NADP**^**+**^**]**	**[NADPH]**	**Anabolic reduction charge**
**Control**	3.07 ± 0.63	0.48 ± 0.08	0.13 ± 0.00	0.61 ± 0.07	0.55 ± 0.17	0.46 ± 0.11
**Inhibitors**	2.26 ± 0.70	0.20 ± 0.06	0.08 ± 0.00	0.37 ± 0.09	0.14 ± 0.01	0.27 ± 0.02

Concentrations are in μmol (g DW)^-1^.

The numbers reported are means from three individual bioreactors ± standard deviation.

### Influence of HMF and furfural on cellular energetics

Similar to the redox co-factors, the adenonucleotides are participating in many intracellular reactions. Furthermore, the concentrations of these compounds can give an indication of the energy state of the cells [37]. To investigate whether continuous stress by furan aldehydes influences the energy balance of the cells, ATP, ADP and AMP were determined at steady-state in both control and step-changed cultivations. In the same way as for the redox co-factors, the metabolism was quenched in methanol at −40°C. The intracellular ATP concentration was found to decrease from 7.36 (±0.36) μmol (g DW)^-1^ to 5.96 (±0.41) μmol (g DW)^-1^ when the feed-medium was step-changed with HMF and furfural, while the concentration of AMP increased from 0.25 (±0.01) to 0.30 (±0.03) μmol (g DW)^-1^. No significant difference could be observed in the intracellular ADP concentration. The energy charge did not vary much between the two cultivations. It remained high, 0.87 (±0.002) and 0.85 (±0.004) for controls and inhibitor-treated cells, respectively (Table 3).

**Table 3:** Intracellular concentrations of ATP, ADP, AMP, energy charge and ATP yield at steady-state from chemostat cultivations with and without HMF and furfural added to the feed-medium

	**[ATP]**	**[ADP]**	**[AMP]**	**E**_**c**_	**Y**_**ATP **_**g DW (mmol ATP)**^**-1**^
**Control**	7.36 ± 0.36	1.87 ± 0.13	0.25 ± 0.01	0.87 ± 0.00	11.0 ± 0.48
**Inhibitors**	5.96 ± 0.41	1.84 ± 0.11	0.30 ± 0.03	0.85 ± 0.00	12.4 ± 0.14

Concentrations are in μmol (g DW)^-1^ unless stated otherwise.

The numbers reported are means from three individual bioreactors ± standard deviation.

Another energy related parameter is the ATP yield (Y_ATP_). This parameter, which relates the amount of formed biomass to the amount of produced ATP, can be estimated from the extracellular concentrations of ethanol, acetate and glycerol, as these metabolites are all accompanied by either generation or consumption of ATP (see methods section). The ATP yield was found to be higher when furan aldehydes were present in the feed-medium. The ATP yield in control cultivations was 11.0 (±0.48) g DW (mmol ATP)^-1^ while it was 12.4 (±0.14) g DW (mmol ATP)^-1^ when furan aldehydes had been added. The results of the quantification of the adenonucleotides and ATP yield are summarized in Table 3.

### Transcriptome response to HMF and furfural stress

To investigate how the cells respond to the presence of HMF and furfural on a global transcriptional level, transcriptome analysis on steady-state samples was performed using DNA arrays. With the thresholds used (false discovery rate (FDR) < 0.01, log_2_fold-change > 0.4), 113 ORFs were found to be up-regulated (Additional file 1) and 121 ORFs were down-regulated (Additional file 2) when HMF and furfural were present in the feed-medium, compared to the control. 17 ORFs were up-regulated more than two-fold and 20 ORFs showed a more than two-fold down-regulation compared to the control. To help interpreting the transcriptional response, the up and down-regulated ORFs were assigned to functional categories using Munich Information Center for Protein Sequences (MIPS) Funcat. The classification revealed that the category “Cell rescue, defense and virulence” was significantly enriched (p < 0.05) in the up-regulated gene set. The ORFs annotated to this class were *PDR5, PDR16, YOR1, LRP1, ALK2, MRK1, GRX3, WWM1, MSB2, CUP1-1, SML1, TIR2, DDP1, SRL1, PAU5* and *PAU7.* The functions of these genes are reported in Table 4.

**Table 4:** ORFs up-regulated in response to HMF and furfural belonging to the MIPS functional category “Cell rescue, defense and virulence”

**Gene name**	**Function**	**log**_**2**_**fold-change**
*PDR5*	Full-size ABC transporter involved in multidrug resistance	1.65
*PDR16*	Protein involved in lipid biosynthesis and multidrug resistance	1.01
*YOR1*	ABC transporter involved in multidrug resistance	0.73
*LRP1*	Like rRNA Processing protein involved in regulation of DNA repair and recombination	0.74
*ALK2*	Strong similarity to DNA damage responsive protein Alk1p	0.45
*MRK1*	Ser/thr protein kinase	1.02
*GRX3*	Member of the subfamily of yeast glutaredoxins	0.60
*WWM1*	WW domain containing protein interacting with Metacaspase Mcp1	0.57
*MSB2*	Multicopy suppressor of a cdc24 bud emergence defect	0.53
*CUP1-1*	Metallothionein	0.45
*SML1*	Protein inhibitor of ribonucleotide reductase	0.45
*TIR2*	Cold shock induced protein	1.68
*DDP1*	Diadenosine hexaphosphate (Ap6A) hydrolase	0.48
*SRL1*	Suppressor of rad53 null mutant lethality	0.54
*PAU5*	Member seriPAUperin multigene family, active during alcoholic fermentation	1.64
*PAU7*	Member of seriPAUperin family	0.99

121 ORFs were found to be down-regulated in response to HMF and furfural. Classification in MIPS functional categories showed that ORFs involved in “Metabolism” (42), “Energy” (22) and “Cellular transport” (25) were significantly enriched. The group related to “Metabolism” contained mostly genes involved in metabolism of the amino acids aspartate, asparagine, cysteine, glycine, tyrosine and alanine. In the group annotated to “Energy”, genes related to the glyoxylate cycle, electron transport and membrane-associated energy conservation were significantly enriched. ORFs enriched in the “Cellular transport” group could be subdivided into groups involved in amino acid/amino acid derivatives transport and electron transport. A remarkable number of amino acid transporters were down-regulated in response to HMF and furfural. This can possibly be connected to the fact that several genes indicating nitrogen starvation were up-regulated. Genes related to pseudohyphal growth are known to be up-regulated during nitrogen-starvation [38], and in fact, the second most up-regulated (log_2_fold-change = 2.20) gene was *MUC1,* encoding a GPI-anchored cell surface glycoprotein required for flocculation and pseudohyphal growth.

As mentioned above, oxidoreductases have been identified as important enzymes in the response to furan aldehydes. In the present study, four genes categorized as oxidoreductases by Gene Ontology (GO) Slim Mapper were up-regulated: *CUP1-1*, *FMS1*, *GRX3* and *HBN1*. *CUP1-1* is a metallothionein that binds copper and mediates resistance to high concentrations of copper and cadmium. *FMS1* encodes a polyamine oxidase converting spermine to spermidine. Spermine and spermidine are polyamines known to protect against abiotic stresses in plants, and in *S. cerevisiae*, spermidine is essential since it functions as substrate for the eukaryote protein synthesis initiation factor eIF5A [39,40]. *GRX3* codes for a glutaredoxin that is a glutathione-dependent oxidoreductase protecting cells from oxidative stress. *HBN1* encodes a putative protein of unknown function similar to bacterial nitroreductases. All of the up-regulated oxidoreductases are located in the cytoplasm. On the other hand, 16 ORFs encoding proteins with oxidoreductase activity were down-regulated. These included *AAD10, ALD5, ERV1, ETR1, GDH2, GTT1, MCR1, MDH2, OYE3, PRX1, SCO1, SDH2, SOD2, UGA2, YAH1* and *YMR226C* (see Additional file 2 for functions). Interestingly, 14 out of these 16 ORFs’ gene products are located in the cytoplasm, but 11 are also annotated to the mitochondria.

It has previously been reported that perturbations in the redox metabolism can induce changes in gene expression thereby reflecting redox sensitive regulation mechanisms [41]. To extend the analysis on how the observed perturbations in the redox co-factors may influence gene expression, the reactions from a genome-scale metabolic model [42] were used to identify metabolic reactions in which NAD(H) or NADP(H) participate as co-factors in order to investigate if any of these genes were differentially expressed as a result of the HMF and furfural-induced redox perturbations. To obtain a less stringent data set, the 1,000 most up-regulated and down-regulated genes from the transcriptome analysis were compared to the identified genes encoding NAD(P)(H)-using enzymes from the genome-scale metabolic model. 8 ORFs which use NAD(H) as co-factor and 6 ORFs that use NADP(H) as co-factor were found among the 1000 most up-regulated genes. One ORF that can use both co-factors was up-regulated. Among the 1000 most down-regulated genes in the data set, 11 used NADP(H) as co-factor, while 12 use NAD(H). 5 ORFs were reported to use both. With the data available in the present study, there is no evidence of any redox-related regulation of NAD(P)H-utilizing enzymes on gene level resulting from the HMF and furfural-induced perturbations in the co-factor levels.

## Discussion

In the present study the physiological effects of HMF and furfural, two inhibitors present in lignocellulosic hydrolysates, were investigated. By having the inhibitors present in the feed-medium of an anaerobic chemostat, physiological responses related to changes in specific growth rate could be circumvented. It has previously been shown that gene expression changes due to alterations in growth rate and the expression pattern observed after exposure to different types of stress overlap to a certain extent [32], which consolidates the choice of operation mode in the present study since HMF and furfural inhibit cell growth at sufficiently high concentrations [29]. In the current experimental setup, a long-term stress response was expected where the cells have adapted to the stressful conditions as compared to the transient stress responses obtained in pulse experiments.

Step-changing the feed-medium by including HMF and furfural resulted in decreased specific uptake-rates for both glucose and xylose compared to the control. Banerjee et al. [3] have reported that furfural inhibits hexokinase, which could have triggered the observed decrease of sugar uptake rates. Although the specific xylose uptake rate decreased, there was an increase in the xylitol production rate and xylitol yield, which suggests that the redox metabolism was perturbed. Xylose consumption in the strain used in the present study occurs via the two oxidoreductases xylose reductase (XR) and xylitol dehydrogenase (XDH) from *S. stipitis*. XR preferably uses NADPH for reduction of xylose to xylitol, while XDH uses NAD^+^ for oxidation of xylitol to xylulose, thereby creating a redox imbalance with xylitol accumulation as a consequence. Previous studies have reported that addition of external electron acceptors such as acetoin, furfural and HMF decrease xylitol accumulation due to regeneration of NAD^+^[43]. To the authors’ best knowledge, one study with a fermentation setup similar to the one described here has been published, i.e. anaerobic chemostat with glucose and xylose in the feed-medium, although only HMF was considered [23]. Two of the strains assessed in that study showed no effect on xylitol yield when the feed was step-changed to an HMF containing one, whereas one strain showed a decreased xylitol yield. The results obtained in the present study can be an effect of the use of both furfural and HMF in the medium. Since it has been demonstrated that the co-factor usage by the detoxifying enzymes is different for HMF and furfural [11], challenging the cells with both these inhibitors simultaneously may perturb the redox metabolism in a different way than reported before.

Even though HMF and furfural detoxification is generally thought to proceed through NAD(P)H-dependent reduction [2], there are surprisingly few reports available on the quantitative effect on the redox co-factor pools and the resulting redox perturbations HMF and furfural leads to. In the present study, we showed that the presence of HMF and furfural in the feed-medium significantly decreased the intracellular steady-state concentrations of NADH and NADPH. Moreover, the catabolic and anabolic reduction charges were decreased significantly. NAD(P)^+^ and NAD(P)H are used as co-factors in numerous intracellular reactions, and consequently, perturbation of the levels of these can have cell-wide effects which can be hard to predict. Most of the intracellular NADH is produced in glycolysis and in biosynthetic reactions such as amino acid synthesis. NADH produced in assimilatory reactions is reoxidized to NAD^+^ by conversion of dihydroxyacetone phosphate to glycerol-3-phosphate by Gpd1p. Addition of external electron acceptors such as HMF and furfural have shown to decrease the glycerol yield by acting as alternative redox sinks. However, in the present study no effect on the glycerol yield was observed after changing to an HMF and furfural containing feed-medium. Assuming an intracellular volume of 2.38 mL (g DW)^-1^[35], the intracellular concentration of NADH decreased from 0.20 mM to 0.08 mM upon inhibitor addition. The K_m_ for NADH of Gpd1p is 0.023 mM [44], thus 4 times lower than the actual concentration in the cell, which would mean that the reaction would still proceed at approximately 80% of V_max_ when inhibitors were present.

Under anaerobic conditions, NADPH is mainly produced in the oxidative part of PPP from glucose-6-phosphate through two reactions catalyzed by Zwf1p and Gnd1p [45]. NADPH is generally used as a reductant in amino acid synthesis and especially in ammonium assimilation through production of glutamate from 2-oxoglutarate by Gdh1p [41]. Glutamate, which is involved in biosynthesis of several amino acids, is a highly connected metabolite in the metabolic network. K_m_ of Gdh1p for NADPH is reported to be 0.033 mM [46]. Using the same assumption on intracellular volume as above, the intracellular concentration of NADPH decreased from 0.23 mM to 0.06 mM after adding HMF and furfural to the feed-medium. With this significant reduction in NADPH concentration, the Gdh1p-catalyzed reaction would proceed at a rate of about 65% of V_max_, a significant decrease in the glutamate production rate, which could have consequences for amino acid production. A lower flux through the ammonia assimilation pathway might signal to the cell that insufficient amounts of nitrogen are available in the form of glutamate, which thereby could lead to a down-regulation of amino acid synthesis. A lower flux through the ammonia assimilation pathway may also be reflected by some features detected by the transcriptome analysis, such as the induction of pseudohyphal growth genes common to the nitrogen starvation response. As many as 10 ORFs encoding amino acid, peptide or amine transporters were identified after functional enrichment analysis of the down-regulated genes in response to HMF and furfural. Several ORFs encoding enzymes involved in amino acid biosynthesis were also found to be down-regulated. Intuitively, one would think that nitrogen starvation and down-regulation of amino acid biosynthesis would lead to up-regulation of amino acid transporters, but surprisingly the opposite was observed. This result can not be easily explained, but it has been observed before in relation to pseudohyphal growth [38].

Since the first step in the xylose-utilization pathway involves an NADPH-dependent reduction, it could be argued that lower intracellular NADPH levels should decrease the flux through the pathway in a similar way as suggested for glutamate synthesis. However, addition of HMF and furfural did not result in a significant decrease in the specific uptake rate of xylose. This could be explained by a comparison of the kinetic properties, and in particular the K_m_ for NADPH of XR and Gdh1p. In fact, the K_m_ of XR for NADPH is 0.009 mM [47], thus 3.7 times lower than that of Gdh1p, which thereby makes XR activity less sensitive to the lower NADPH levels observed after HMF and furfural addition.

NAD(P)H dependent reduction of HMF and furfural to their corresponding alcohols requires the supply of sufficient amounts of the involved co-factors. In a recent study [48], it was shown that increased NADPH demand caused by addition of 200–300 mM of the electron acceptor acetoin induced the expression of genes involved in NADPH generation, such as *GND1* in the oxidative part of PPP*.* In contrast, the higher co-factor demand triggered by lower concentrations (100 mM) of acetoin could be met through metabolic (post-transcriptional) regulation. Similarly to this latter case, addition of HMF and furfural did not induce expression of genes involved in NADPH synthesis in the present study. Thus, the higher demand for redox co-factors in the presence of furan aldehydes in the present study is possibly regulated at metabolic level, which in fact was the case when the lower amount (100 mM) acetoin was added to the cultivations in [48].

Interestingly, the energy charge did not change notably and remained at a high level after addition of furaldehydes to the feed-medium. On the other hand, the biomass yield (Y_SX_) and the ATP yield (Y_ATP_) $\left(\mathrm{defined}\;\mathrm{as}\;\frac{\left[\mathrm{Biomass}\right]}{\left[\mathrm{Ethanol}\right]+\left[\mathrm{Acetate}\right]-\left[\mathrm{Glycerol}\right]}\right)$ increased when HMF and furfural were present. The energy requirement for biomass formation, or Y_ATP_, is calculated as the amount of biomass produced in relation to the amount of ATP produced, and can therefore be seen as a coupling factor between anabolism and catabolism [49]. Protein synthesis, ammonium transport and amino acid synthesis are three of the most ATP consuming processes in metabolism [49]. A decrease in protein polymerization and/or alternatively, a decreased amino acid production could in fact provide more ATP available for carbon assimilation. Thus, changes in biomass composition can have a considerable impact on the biomass yield and ATP yield.

An alternative explanation for the increase in biomass yield that has been proposed [23] is that reduction in glycerol production can lead to an increased amount of ATP available for biomass production, since glycerol production is an ATP consuming process. NADH generated in the biosynthesis of amino acids can be reoxidized to NAD^+^ through NADH-dependent reduction of HMF and furfural instead of glycerol production, which is the native redox valve. In the present study, the specific glycerol production rate decreased when HMF and furfural were present in the feed-medium, but the difference was not statistically significant.

Functional enrichment of the ORFs that were up-regulated after a step-change to HMF and furfural containing feed-medium showed that only one functional class (Cell rescue, defense and virulence) was significantly over-represented. Two of the ORFs in this class, *PDR5* and *YOR1*, are coding for ATP-binding cassette (ABC) transporters that function in efflux of several compounds, including ions and xenobiotics. They are under transcriptional control of Pdr1p and Pdr3p, and can probably function in transporting either HMF and furfural, or their corresponding alcohols, out of the cell, thereby relieving the stress caused by these agents. The lower intracellular ATP concentration that was observed after HMF and furfural had been added could be an indication that ATP was used by the efflux pumps in order to transport the inhibitors out of the cell. In fact, Ma et al. [31] constructed *Δpdr1* and *Δpdr3* mutants and showed that these strains exhibited a longer lag-phase than the wild-type in the presence of HMF, indicating that ABC transporters are important for tolerance to furan aldehydes. Moreover, Alrikson et al. [50] overexpressed *FLR1* and *ATR1*, which are multidrug transporters of the major facilitator family, and obtained strains with higher tolerance to HMF and coniferylaldehyde, respectively. Together with our results, these examples show that multidrug transporters are putative metabolic engineering targets for increased tolerance to HMF and furfural.

Finding the molecular mechanisms of the inhibitory action by detailed physiological analyses is a promising strategy to discover metabolic engineering targets that can improve strain robustness. HMF and furfural remain a serious challenge for lignocellulosic ethanol production. Not least since attainment of higher final ethanol titers requires that higher solid loadings are used in the production process, which comes at the cost of higher inhibitor concentrations [51]. From the results of the physiological analyses in the present study it is clear that the redox metabolism is severely affected by furan aldehydes, while the effects on the energetics is less evident. Thus, engineering of the redox system represents a putative target to relieve the stress caused by HMF and furfural, but further studies are required to target the specific consequences of the perturbed redox metabolism.

## Conclusion

We demonstrated that HMF and furfural perturbed the redox system of xylose-utilizing *S. cerevisiae,* which is likely to have serious consequences for metabolism, and may constitute the molecular mechanism of action. Targeting redox metabolism can hence be a successful strategy to overcome inhibition and lead to development of more robust yeast strains for lignocellulosic ethanol production.

## Methods

### Cell culture

#### Strain

The strain investigated in this study, VTT C-10883, was derived from VTT C-10880 *(MAT*α, *MAL2-8c, SUC2 ura3::XYL1 XYL2, XKS1::XKS1).* VTT C-10880 was obtained in the genetic background of CEN.PK 113-1A (*MAT*α, *MAL2-8c, SUC2)* by overexpressing the endogenous xylulokinase encoding gene (*XKS1*) and integrating *S. stipitis* XR (*XYL1*) and XDH (*XYL2*) encoding genes in the *URA3* locus [52]. Prototrophy was restored by integrating an intact copy of *URA3* in 5′ of the heterologous construct and the resulting strain was named VTT C-10883.

#### Preparation of inoculum

Inoculum cultures were prepared from −80°C glycerol stocks on agar plates containing 10 g L^-1^ yeast extract, 20 g L^-1^ soy peptone, 20 g L^-1^ agar and 20 g L^-1^ glucose. Colonies were transferred to 100 mL medium in a 500 mL Erlenmeyer flask. The composition of the medium was 20 g L^-1^ glucose, 5 g L^-1^ (NH_4_)_2_SO_4_, 3 g L^-1^ KH_2_PO_4_, 0.5 g L^-1^ MgSO_4_⋅7H_2_O, 1 mL L^-1^ vitamin solution, 1 mL L^-1^ trace metal solution. The vitamin and trace metal solutions were prepared according to [53]. The cultures were grown for 24 hours at 30°C and pH 5 in a rotary shaker at 150 rpm.

#### Anaerobic chemostat cultivations

Precultured cells were inoculated to an OD_600_ of 0.2 in three individual bioreactors (DASGIP AG, Jülich, Germany) in a final working volume of 300 mL. The medium was sparged with 0.2 VVM of nitrogen to maintain anaerobic conditions. The medium had the same composition as for the preculture with the exception that ergosterol and Tween 80 were added at a final concentration of 0.01 g L^-1^ and 0.42 g L^-1^, respectively, to allow anaerobic growth. The batch phase was finished after approximately 13 hours (as observed on the drop in the on-line measured carbon dioxide evolution rate) after which the feed to the reactor was started. The feed-medium had the same composition as the medium for the batch phase, except as carbon source a mixture of 20 g L^-1^ glucose and 50 g L^-1^ xylose were used. The dilution rate was set to 0.1 h^-1^ and the cultivation was judged to be at steady state after the carbon dioxide evolution rate had been constant for at least 4 residence times. The cultivation was maintained at 30°C and pH 5 by automatic addition of 1 M NaOH.

### Analysis of extracellular metabolites

Extracellular metabolite samples taken from the chemostat cultivations were filtered through 0.2 μm syringe-filters (VWR International, West Chester, US). The samples were stored at −20°C until analysis. Analysis of sugars and metabolites were performed using a HPLC system (Ultimate 3000, Dionex, Sunnyvale, US). Glucose, xylose, ethanol, xylitol, glycerol, acetic acid, HMF and furfural were separated using an Aminex HPX87-H column (Bio-Rad Laboratories, München, Germany) with 5 mM H_2_SO_4_ as eluent. The column was operated at 60°C and at a flow rate of 0.6 mL min^-1^. Ethanol, xylitol, glycerol and acetic acid were detected with a refractive index detector Shodex RI-101 (Showa Denko, New York, NY) while HMF and furfural were detected using an UV detector at 210 nm (Dionex, Sunnyvale, US).

### Determination of cell mass

The dry cell mass was determined by filtering 5 mL of culture broth through pre-dried 0.45 μm PES membranes (Sartorius Stedim, Aubagne, France). The filters were washed with MilliQ-water and dried in a microwave oven at 120 W for 15 minutes. The filters were left to cool and dry further in a desiccator over night and were subsequently weighed.

### Sampling for intracellular metabolites

#### Quenching

Samples of 5 mL were taken for intracellular metabolites and quenched according to [54] in 25 mL pure methanol in pre-weighed tubes maintained at −40°C. The samples were allowed to cool for three minutes, after which the sample volumes were determined by weighing. The cells were then pelleted in a centrifuge (SIGMA Laborzentrifugen GmbH, Osterode, Germany) maintained at −20°C at 4000 g for 5 minutes. Samples to be used for quantification of NAD(P)H were extracted instantly whereas the other samples were flash-frozen in liquid nitrogen and stored at −80°C until analysis.

#### Extraction of NAD(P)H

The reduced redox-factors are stable in alkaline conditions and were extracted as described in [55]. In short, 0.5 mL of 17% (v/v) alcoholic 1 M KOH was added to the samples, after which they were incubated at 70°C for 7 minutes in a water-bath (Grant Instruments, Shepreth, United Kingdom).

#### Extraction of NAD(P)^+^

NAD^+^ and NADP^+^ were extracted by first suspending the cell pellets in 14% (w/w) HClO_4_ and thereafter disintegrating the cells with glass beads (diameter: 425–600 μm) in a bead mill. The acidic extracts were thereafter neutralized with 2 M KOH supplemented with 0.4 M imidazole.

#### Extraction of ATP, ADP and AMP

ATP, ADP and AMP were extracted according to [56]. After adding 0.5 mL of 0.52 M trichloroacetic acid (TCA) containing 17 mM EDTA the samples were incubated on ice for 15 minutes. The extracts were then centrifuged at 14 000 rpm for 3 minutes and subsequently neutralized with 2 M Tris-base.

### Analysis of intracellular metabolites

#### Quantification of NAD(P)^+^ and NAD(P)H

NADH and NADPH were first oxidized enzymatically since the reduced co-factors are unstable at non-alkaline conditions. The reaction was performed by adding 6 μL glutamate dehydrogenase (GlDH, 240 U mL^-1^) to extracts neutralized with substrate/buffer mixture containing (5 mM 2-oxoglutarate, 0.5 M HEPES, 0.5 M phosphate and 30 mM NH_4_Cl). The reaction took place at room temperature and was stopped with 3 M HClO_4_ after 20 minutes. The extracts were subsequently neutralized with 3 M KOH. The redox co-factors were quantified by enzymatic cycling according to [35,57] for NADH and NADPH, respectively, modified for use in 96-well plates. The assay mixture for determination of NAD^+^ contained 2 mM phenazine ethosulfate (PES), 0.5 mM thiazolyl blue (MTT), 120 mM bicine, 1.61 M ethanol and 50 μL sample in 300 μL total reaction volume. The assay mixture for determination of NADP^+^ contained 2 mM PES, 0.5 mM MTT, 120 mM bicine, 12.7 mM glucose-6-phosphate, 4.2 mM MgSO_4_⋅7H_2_O and 50 μL sample. The reactions were initiated by adding 5 μL alcohol dehydrogenase (ADH, 231 U mL^-1^) for determination of NAD^+^ or 5 μL glucose-6-phosphate dehydrogenase (G6PDH, 560 U mL^-1^) for determination of NADP^+^. The rate of formation of formazan, which correlates with the concentration of NAD(P)^+^, was measured at 570 nm in a plate reader (BMG Labtech GmbH, Ortenberg, Germany). Both reactions were performed at 30°C and standards of known concentrations were used for quantification.

#### Quantification of ATP, ADP and AMP

Concentrations of adenonucleotides were determined via HPLC (Ultimate 3000, Dionex, Sunnyvale, US) equipped with a quaternary analytical pump (HPG-3400A, Dionex, Sunnyvale, US) fitted with a Luna® 5u C18(2) 100 Å LC column (150 x 4.6 mm) (Phenomenex Inc., Torrance, US) kept at 20°C. The mobile phase consisted of acetonitrile (A) and tetrabutylammonium buffer (B) (0.005 M tetrabutylammonium hydrogensulfate, 0.01 M Na_2_HPO_4_), pH 7.0. The pump was programmed to generate the following gradient: 6% A and 94% B (0 to 3 min), a linear increase of A to 25% and a linear decrease of B to 75% (3 to 16 min), 25% A and 75% B (16 to 22 min), a linear decrease of A to 6% and a linear increase of B to 94% (22 to 27 min) and 6% A and 94% B (27 to 35 min). The flow rate was 1 mL min^-1^. The detection was performed with a photodiode array detector (PDA-3000, Dionex, Sunnyvale, US) at 260 nm. Peak identities were confirmed by co-elution with standards and quantification was carried out by comparison using standard solutions of known concentrations.

### Transcriptome analysis

5 mL samples withdrawn from three individual bioreactors for each condition were rapidly cooled on ice in pre-chilled Falcon tubes after which they were centrifuged at 4000 g for 2 minutes. The cell pellets were frozen in liquid nitrogen and stored at −80°C until analysis.

The cells were mechanically disintegrated in a bead mill and total RNA was extracted and purified with RNeasy Mini Kit (QIAGEN) according to the manufacturer’s instructions. The integrity of the RNA was assessed with an Agilent 2100 Bioanalyzer. Labelled RNA was produced using the GeneChip® 3^′^ IVT Express Kit (Affymetrix) after which the labelled RNA was hybridized onto GeneChip® Yeast Genome 2.0 arrays. Three chips were hybridized for each condition. Staining and washing was performed in a GeneChip® Fluidics Station 450 (Affymetrix) and scanning of the chips were carried out in a GeneChip® Scanner 3000 7 G (Affymetrix).

Data analysis was performed with the Bioconductor R package (version 2.8). The raw intensity data was normalized with quantiles, background corrected with the gcrma algorithm and summarized using median polish. Differentially expressed ORFs were assessed using the limma package in R [58]. ORFs with a Benjamini-Hochberg false discovery rate lower than 0.01 were considered statistically significant and only ORFs with a log_2_fold-change greater than ±0.4 were taken into account. Enriched functional categories were found with the MIPS functional catalogue (http://mips.helmholtz-muenchen.de/proj/funcatDB) and GO slim mapper of the *Saccharomyces cerevisiae* genome database (SGD) (http://www.yeastgenome.org).

### Calculations

The yields were calculated based on the amount of consumed sugars (glucose and xylose) and statistical significance was determined with Student’s *t*-test in Microsoft Excel 2011.

The ATP yield was determined from equation 1:

(1) $$Y_{\mathrm{ATP}}=\frac{\left[\mathrm{Biomass}\right]}{\left[\mathrm{Ethanol}\right]+\left[\mathrm{Acetate}\right]-\left[\mathrm{Glycerol}\right]}$$

The energy charge was calculated from equation 2:

(2) $$E_{c}=\frac{\left[\mathrm{ATP}\right]+0.5\times \left[\mathrm{ADP}\right]}{\left[\mathrm{ATP}\right]+\left[\mathrm{ADP}\right]+\left[\mathrm{AMP}\right]}$$

The catabolic and anabolic reduction charges were calculated from equations 3 and 4, respectively:

(3) $$\frac{\left[\mathrm{NADH}\right]}{\left[\mathrm{NADH}\right]+\left[\mathrm{NA}D^{+}\right]}$$

(4) $$\frac{\left[\mathrm{NADPH}\right]}{\left[\mathrm{NADH}\right]+\left[\mathrm{NAD}P^{+}\right]}$$

## Competing interests

The authors declare that they have no competing interests.

## Authors’ contributions

MA participated in the design of the study, performed the experiments and drafted the manuscript. MB participated in the design of the study, participated in the sampling of the intracellular metabolites and helped to draft the manuscript. VM developed the protocol for adenonucleotide quantification and commented on the manuscript. LO participated in the design of the study and commented on the manuscript. All authors reviewed and approved the final manuscript.

## Supplementary Material

Additional file 1 **ORFs up-regulated in response to HMF and furfural.** Table showing all significantly up-regulated ORFs (FDR < 0.01, log_2_fold change >0.4) and functional descriptions. Click here for file

Additional file 2 **ORFs down-regulated in response to HMF and furfural.** Table showing all significantly down-regulated ORFs (FDR < 0.01, log_2_fold change < −0.4) and functional descriptions. Click here for file
